# Repurposing of Pirfenidone (Anti-Pulmonary Fibrosis Drug) for Treatment of Rheumatoid Arthritis

**DOI:** 10.3389/fphar.2021.631891

**Published:** 2021-03-05

**Authors:** Donghao Gan, Wenxiang Cheng, Liqing Ke, Antonia RuJia Sun, Qingyun Jia, Jianhai Chen, Jietao Lin, Jian Li, Zhanwang Xu, Peng Zhang

**Affiliations:** ^1^ Shenzhen Institutes of Advanced Technology, Chinese Academy of Sciences, Shenzhen, China; ^2^ First College of Clinical Medicine, Shandong University of Traditional Chinese Medicine, Jinan, China; ^3^ Second Ward of Trauma Surgery Department, Linyi People’s Hospital, Linyi, China; ^4^ University of Chinese Academy of Sciences, Beijing, China; ^5^ Department of Orthopedics, Affiliated Hospital of Shandong University of Traditional Chinese Medicine, Jinan, China; ^6^ Shenzhen Engineering Research Center for Medical Bioactive Materials, Shenzhen, China

**Keywords:** pirfenidone, rheumatoid arthritis, collagen-induced arthritis, fibroblast-like synoviocytes, endothelial cell

## Abstract

Clinical studies have shown that pirfenidone (PFD) effectively relieves joint pain in rheumatoid arthritis (RA) patients. However, the detailed mechanisms underlying the anti-RA effects of PFD have not been investigated. This study was undertaken to investigate the repurposing of PFD for the treatment of RA, and explore its anti-rheumatic mechanisms. A collagen-induced arthritis (CIA) rat model was used to observe joint pathological changes following PFD treatment. Based on bioinformatics to predict the mechanism of PFD anti-RA, using EA. hy926 and TNF-α-induced MH7A cells to establish *in vitro* model to explore its biological mechanism from the perspectives of synovial inflammation and angiogenesis. PFD significantly relieved pathological changes, including joint swelling, synovial hyperplasia, inflammatory cell infiltration and joint destruction. PFD was also associated with reduced expression of MMP-3 and VEGF in articular chondrocytes and synovial cells of CIA rats (*p* < 0.05). Using bioinformatic methods, we predicted that PFD inhibits cell inflammation and migration by interfering with the JAK2/STAT3 and Akt pathways. These results were verified using *in vitro* models. In particular, PFD effectively reduced the expression of pro-inflammatory, chondrogenic, and angiogenic cytokines, such as IL-1β, IL-6, IL-8, MMP-1/3/2/9 and VEGF (*p* < 0.05), in TNF-α-induced MH7A cells. In addition, PFD significantly reduced the production of MMP-2/9 and VEGF in EA. hy926 cells, thereby weakening migration and inhibiting angiogenesis (*p* < 0.05). These findings suggest that PFD may alleviate the pathological process in CIA rats, by inhibiting inflammation and angiogenesis through multiple pathways, and serve as a potential therapeutic drug for RA.

## Introduction

Rheumatoid arthritis (RA) is a chronic inflammatory joint disease characterized by synovial inflammation, pannus hyperplasia, and cartilage destruction in joints (McInnes and Schett, 2011). Current disease-modifying anti-rheumatic drugs and biological agents used to alleviate the clinical symptoms of RA have obvious adverse effects and are expensive (Guo et al., 2018b). Research recent has focused on treatment strategies targeting synovial inflammation and angiogenesis (Thairu et al., 2011; Veale et al., 2017), as synovial cells participate in almost all pathological processes of RA (Asif Amin et al., 2017), including inflammation and bone destruction, and the pannus is closely related to the progressive destruction of joints (MacDonald et al., 2018; Narayan et al., 2018).

Compared with the development of new drugs, novel applications of established drugs could reduce risks and have obvious advantages for the treatment of refractory diseases (Baek et al., 2015). For example, based on protein interactions, several clinical drugs have been evaluated for the treatment of COVID-19, avoiding time-consuming and expensive drug development (Gordon et al., 2020). Pirfenidone (PFD) could effectively inhibit fibroblast proliferation, alleviate local inflammatory cell infiltration, and reduce collagen deposition *in vivo* (Nakazato et al., 2002; Kakugawa et al., 2004). Moreover, it could regulate wound healing and inhibit endothelial cell angiogenesis *in vitro* (Liu et al., 2017). As PFD has both anti-inflammatory and anti-angiogenic effects, we speculate that it is a potential targeted anti-RA drug. To the best of our knowledge, only one study has reported that PFD could effectively relieve the symptoms of RA and reduce joint pain (Pesce et al., 2004). However, the pathological and regulatory mechanisms underlying the anti-RA effects of PFD have not been investigated so far.

In this study, we investigated the effects of PFD on synovial inflammation and joint destruction in a collagen-induced arthritis (CIA) rat model. Furthermore, based on reported effects of PFD and bioinformatic analyses (Grammer and Lipsky, 2017), we predicted its targets and mechanisms, followed by *in vitro* analyses of MH7A and EA. hy926 cells from the perspectives of both anti-angiogenic and anti-inflammatory effects.

## Materials and Methods

### Materials and Reagents

Anti-p-JAK2 (Cat. no. 3771S), anti-JAK2 (Cat. no. 3230S), anti-p-STAT3(Cat. no. 9145S), anti-STAT3 (Cat. no. 9139S), anti-MMP-2 (Cat. no. 40994S), anti-p65 (Cat. no. 4764S) and anti-p-AKT (Cat. no. 9275S) primary antibodies and HRP-linked secondary antibody (Cat. no. 7074) were purchased from CST (United States). Anti-VEGF (Cat. no. ab46154), anti-AKT (Cat. no. ab32505), and anti-MMP-3 (Cat. no. ab53015) antibodies and DAB substrate kit (Cat. no. ab64238) were obtained from Abcam (United Kingdom). Pirfenidone (Cat. no. S2907) was purchased from Selleckchem (Shanghai, China). Matrigel (Cat. no. 354234) and Transwell (Cat. no. 3422) were purchased from Corning. Recombinant human TNF-α (Cat. no. 10602-HNAE) and recombinant human TGF-β1 (Cat. no. 10804-HNAC) were purchased from Sino biological (Beijing China). Anti-MMP-9 (Cat.no. 10375-2-AP) and GAPDH (Cat.no. 60004-1-Ig) primary antibodies were obtained from Proteintech (Wuhan, China). The H&E staining kit (Cat.no. C0105) was purchased from Beyotime (Shanghai, China). Enzyme-linked immunosorbent assay (ELISA) kits, including IL-8 (D8000C), IL-6 (D6050) and IL-1β (DLB50), and VEGF (DVE00) were purchased from R&D systems (United States). Incomplete Freund’s adjuvant (Cat.no. 7002) and Bovine type II collagen (20,022) were purchased from Chondrex (United States). Chemiluminescent HRP substrate (Cat.no. WBKLS0500) was purchased from Millipore (United States).

### Animal Model of Collagen-Induced Arthritis

A total of 24 male Lewis rats (8 weeks old) were purchased from Beijing Vital River Laboratory Animal Technology (Beijing, China). The animals were housed in a specific pathogen-free facility at the Peking University Shenzhen Graduate School. The experimental protocol was approved by the Laboratory Animal Ethical and Welfare Committee of the Shenzhen Institutes of Advanced Technology, Chinese Academy of Sciences (no: SIAT-IACUC-190723-KYC-ZP-A0804). The CIA model was established according to a previous protocol (Brand et al., 2007).

### Treatment and Assessment of Arthritis

A total of 24 rats were randomly divided into four groups (6 rats/group): a normal group (Con) and three CIA models. After the second immunization, Most of 24 rats were successfully induced arthritis, then we randomly divided them into three groups with six rats in each group, to ensure that there was no significant difference in the degree of hindfoot swelling and the incidence of arthritis in each group. Then rats in group 2 were administered carboxymethyl cellulose sodium (CMC-NA), 10 ml/kg/day intragastrically (CIA); rats in group 3 were given MTX, 0.1 mg/kg/3 days intraperitoneally (CIA-MTX); and rats in group 4 were administered oral PFD, 500 mg/kg/d intragastrically (CIA-PFD) (Kakugawa et al., 2004), with continuous gavage for 21 days. During the treatment period, the thickness of the posterior paw of the rats was measured every 7 days.

### Micro-Computed Tomography (Micro-CT)

Forty-two days after the establishment of the model, rats were euthanized, the right knee joints were fixed with 4% paraformaldehyde for 1 day, and a micro-CT (SkyScan1176) scan was used to estimate the structural status. The segmented images were 3D-reconstructed, and 1.5-mm sections, extending from the subchondral bone of the tibia to the distal end, were selected for analyses of the BV/TV ratio. The evaluation criteria for joint damage were as follows: 0 = no damage, 1 = minor, 2 = moderate, and 3 = severe (Jia et al., 2019).

### Histomorphometric Analysis

After the rats were euthanized, the knee joints were fixed with 4% paraformaldehyde, decalcified with 10% EDTA for 40 days, and subjected to routine dehydration, paraffin embedding, and 5 µm routine sectioning. Sections were stained with hematoxylin and eosin (H&E) and toluidine blue (TB) to analyze synovial inflammation and cartilage erosion; semi-quantitative evaluation criteria were based on a previously described protocol (Camps et al., 2005; Guo et al., 2018a).

### Ultrasound Analysis

After 42 days, the knees of the rats were examined using the Vevo 2100 Imaging System (Vevo LAB, Toronto, Canada). Scanning was performed in B-mode, and 2D images were analyzed using the Vevo Laboratory software.

### Immunohistochemical Analysis

After routine dewaxing and rehydration, the paraffin-embedded knee sections were subjected to antigen retrieval, hydrogen peroxide incubation, immune blocking, incubation with the primary antibody and secondary antibody, DAB color development, dehydration, and tablet sealing.

### Bioinformatic Analysis of the Anti-RA Effects of PFD

The TTD (http://db.idrblab.net/ttd/) and DisGeNET (https://www.disgenet.org/) databases were used to retrieve targets related to RA, and target symbols were converted into UniProt (https://www.uniprot.org/) IDs for subsequent analyses. The targets were integrated, deduplicated, and imported into the DAVID (https://david-d.ncifcrf.gov/) database for a KEGG enrichment analysis. The STITCH (http://stitch.embl.de/), Swiss TargetPrediction (http://www.swisstargetprediction.ch/), and SuperPred (http://prediction.charite.de/) databases were used to predict the targets of PFD based on ligand structure. These targets were integrated and imported into the String (https://string-db.org/) database to generate a protein-protein interaction (PPI) network, and subnetwork protein functions were analyzed. The putative targets of PFD were expanded using BioGenet and mapped to the RA-related targets. Then, the mapping targets were imported into CytoNCA to obtain hub nodes topologically according to the target degree. Next, a hub node interaction network was constructed, and targets were compared with the predicted targets of PFD. Finally, the hub nodes were imported into the DAVID database for a KEGG enrichment analysis.

### Cell Culture

The human RA-fibroblast-like synoviocyte (FLS) cell line MH7A was purchased from the Riken Cell Bank (Tsukuba, Japan). The human umbilical vein endothelial cell line EA. hy926 was donated by the research group of Professor Qin Ling of the Chinese University of Hong Kong. MH7A and EA. hy926 cells were separately cultured in 1,640 medium or H-DMEM, containing 100 IU/ml penicillin/streptomycin and 10% heat-inactivated fetal bovine serum, in an incubator with 5% CO_2_ at 37°C.

### Cell Viability Assay

An equal amount of cell suspension was placed in a 96-well plate with PFD at various concentrations. The culture plate was incubated in an incubator (37°C, 5% CO_2_) for 72 h, and CCK-8 solution was added to each well and incubated briefly. Absorbance was measured at 450 nm using the Thermo Scientic-Multiskan Sky microplate reader, and the safe application dose of PFD was determined.

### Tube Formation Assay

Matrigel was thawed on ice, spread on a 24-well plate, and incubated in an incubator for 30 min. Equal amounts of the EA. hy926 cell suspension and different concentrations of PFD were then added to each well and incubated for 6 h. Calcein was added and images were subsequently obtained using a fluorescence microscope. The tube effect was analyzed using ImageJ (NIH, Bethesda, MD, United States).

### Scratch Assay

After EA. hy926 cells reached 90% confluence, a 200 µL pipette was used to vertically scratch the dish, followed by washing and the addition of serum-free medium containing different concentrations of PFD. Images were obtained after 24 h, and cell migration was analyzed using ImageJ (Liang et al., 2007).

### Transwell Assay

EA.hy926 cells were inoculated into the upper chamber of a Transwell membrane and PFD was added to the lower chamber; the upper chamber included serum-free medium and the lower chamber included medium containing 2% FBS. After incubation for 4 h, the cells in the upper chamber were wiped with a cotton swab. The cells were fixed with 4% paraformaldehyde and stained with crystal violet. Then, the cells that migrated through the membrane were counted under an inverted light microscope.

### Western Blot Analysis

After protein extraction in a 6-well plate, a BCA assay was used to detect the protein concentration. Electrophoresis, membrane transfer, immunoblocking, incubation with primary and secondary antibodies, and development were performed, and equal protein loading was controlled using GAPDH. ImageJ was used to quantify the gray values of fluorescent bands.

### Quantitative Reverse-Transcriptase PCR Analysis

RNA was extracted from cells treated with PFD in a 6-well plate on ice. The concentration was measured and target gene primers were added (Table 1). Then each sample was reverse transcribed using the cDNA synthesis kit according to the manufacturer’s protocol. The thermocycling condition: 95°C for 30 s→95°C for 5 s→60°C for 30 s→95°C for 5 s, 60°C for 60 s, 97°C for 1 s, 30 cycles, and stored at 4°C. Target gene expression was evaluated using the ^△△^Ct method after reverse transcription and amplification.

**TABLE 1:** Sequences of the primers.

Name	Forward	Reverse
IL-1β	TCC GAC CAC CAC TAC AGC AAG G	GGA GCG TGC AGT TCA GTG ATC G
IL-6	GGT GTT GCC TGC TGC CTT CC	GCT CTG GCT TGT TCC TCA CTA CTC
IL-8	GGT GCA GTT TTG CCA AGG AG	TTC CTT GGG GTC CAG ACA GA
MMP-1	CTC AAT TTC ACT TCT GTT TTC TG	CAT CTC TGT CGG CAA ATT CGT
MMP-3	GGC TTC AGT ACC TTC CCA GG	GCA GCA ACC AGG AAT AGG TT
MMP-2	CTT CCA AGT CTG GAG CGA TGT	TAC CGT CAA AGG GGT ATC CAT
MMP-9	CCT CTG GAG GTT CGA CGT GA	TAG GCT TTC TCT CGG TAC TGG AA
VEGF	GAG CCT TGC CTT GCT GCT CTA C	CAC CAG GGT CTC GAT TGG ATG
GAPDH	GGA GTC CAC TGG CGT CTT	AGG CTG TTG TCA TAC TTC TCA T

### Enzyme-Linked Immunosorbent Assay

The cell culture fluid was supplemented with PFD in a 6-well plate, followed by centrifugation according to the manufacturer’s protocol, and the supernatant was collected for protein detection according to the manufacturer’s instructions.

### Statistical Analysis

Each group of experiments was repeated three times independently, and data are expressed as means ± standard deviation (SD). GraphPad Prism 8.0 was used for one-way analysis of variance. P-values less than 0.05 and 0.01 were considered significant.

## Results

### PFD Attenuates Pathological Changes in the CIA Rat Model

**FIGURE 1:**
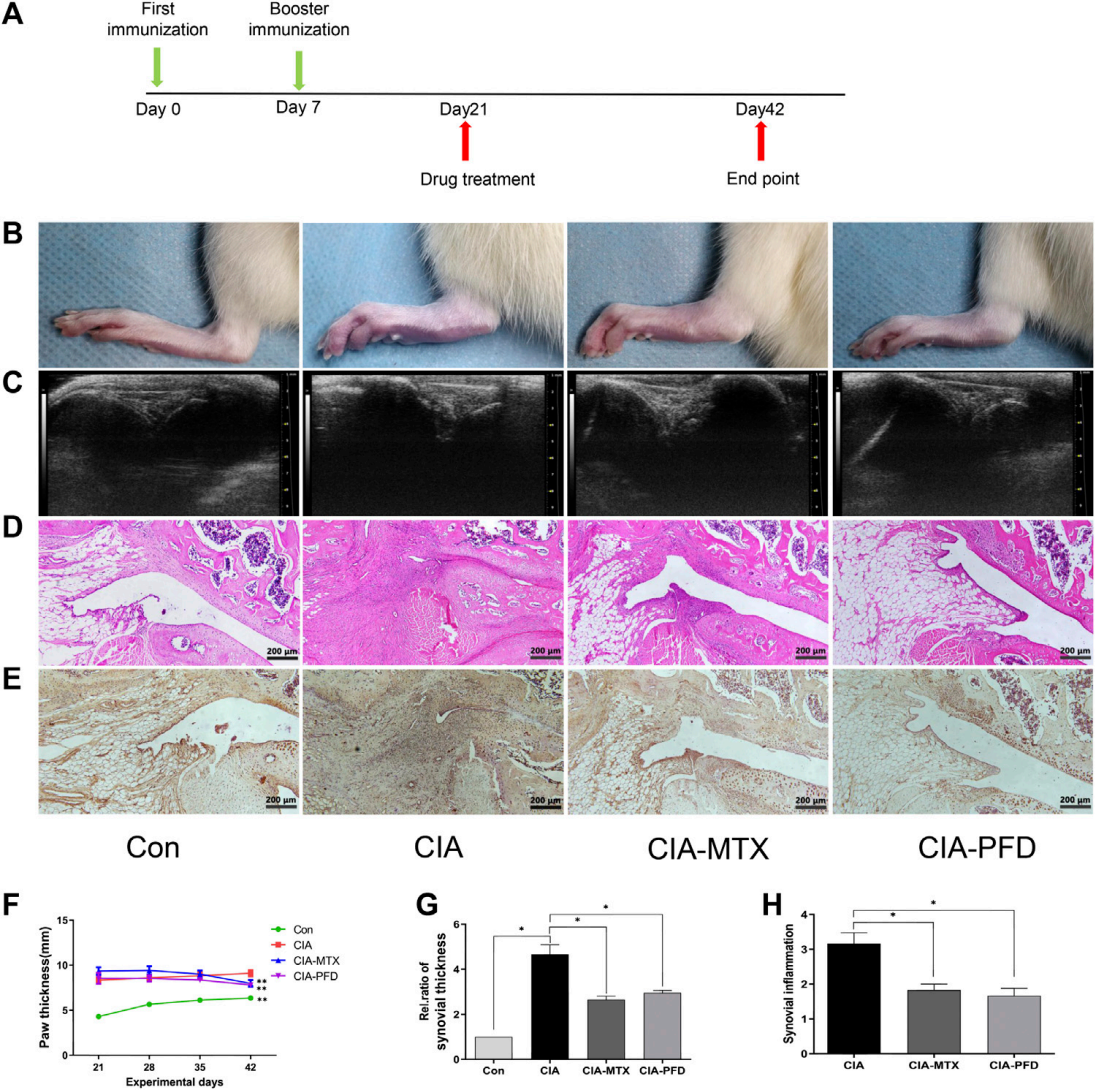
PFD attenuated symptoms and synovitis in CIA rats and reduced the expression of VEGF in the knee joints **(A)** Flow chart of the experimental design. **(B)** Representative images of the hind paws of CIA rats **(C)** B ultrasonic images of the knee joint in the anteroposterior position. **(D)** H&E staining was used to analyze pathological changes of the knee joints **(E)** Immunohistochemical staining results for VEGF. **(F-H)** Semiquantitative data are shown as means ± SD of six rats per group. *p < 0.05. *In vivo* experimental data are indicated by gray coloring in the figures.

Compared to the normal group, CIA rats had greater hindfoot paw thickness, and both MTX and PFD treatments reduced hindfoot swelling (*p* < 0.05) (Figures 1B,F). As determined with an ultrasound analysis, synovial thickness of the knee joint was significantly lower in the CIA rats treated with PFD than in the untreated CIA rats (*p* < 0.05) (Figures 1C,F). H&E staining showed that, compared with the CIA group, PFD significantly alleviated synovial hyperplasia and inflammatory cell infiltration in the knee joints (*p* < 0.05) (Figures 1D,F); immunohistochemistry also indicated that PFD effectively reduced VEGF protein expression in CIA joints (Figure 1E).

**FIGURE 2:**
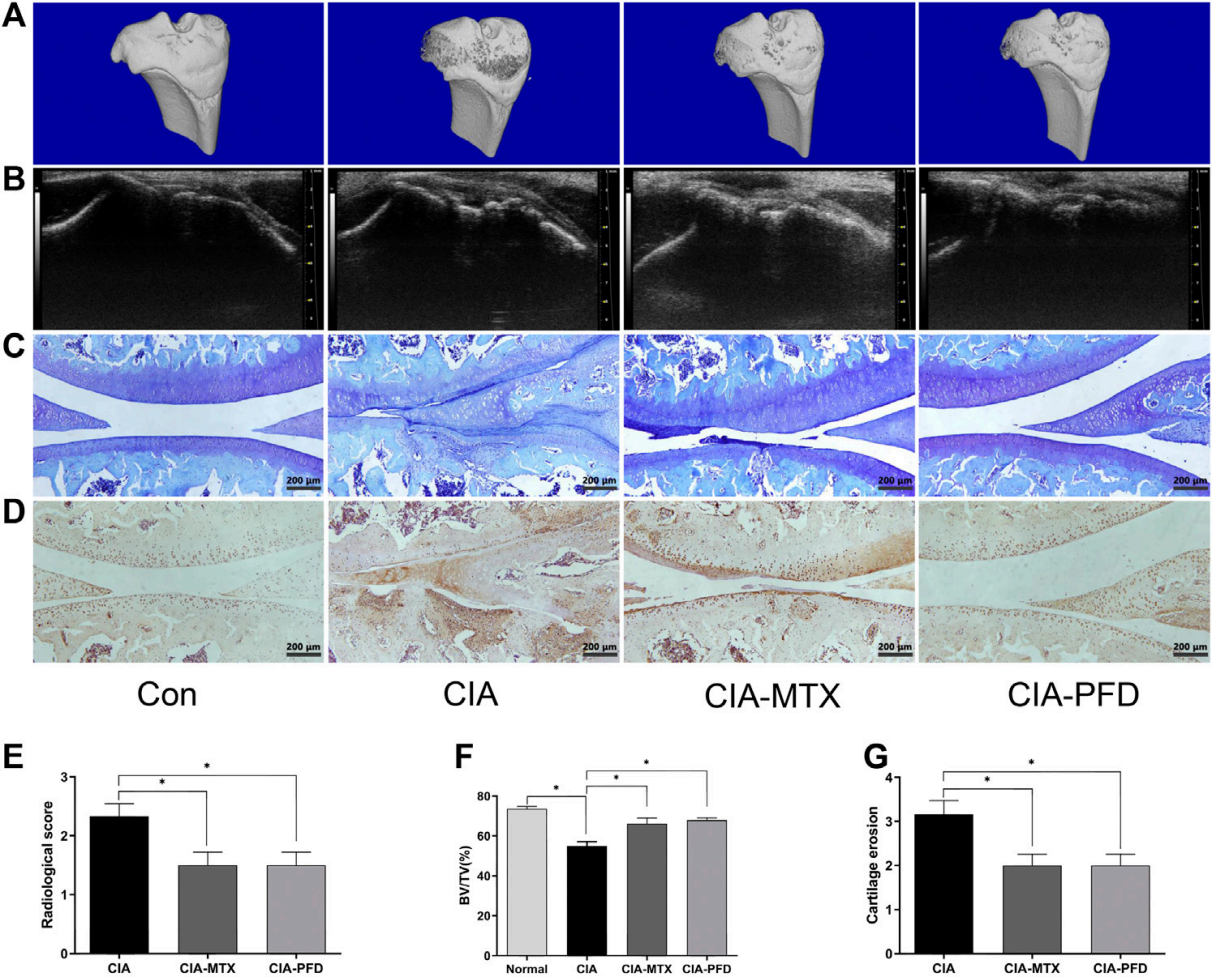
PFD alleviated joint destruction and reduced the expression of MMP-3 in the knee joint of CIA rats **(A)** 3D reconstruction of the tibia by micro-CT. **(B)** B ultrasonic images in the lateral position **(C)** Toluidine blue staining of articular cartilage. **(D)** Immunohistochemical staining results for MMP-3. **(E-G)** Semiquantitative data are shown as means ± SD of six rats per group. *p < 0.05. *In vivo* experimental data are indicated by gray coloring in the figures.

The articular cartilage and subchondral bone of CIA rats were damaged, and PFD significantly alleviated the joint damage in CIA rats (*p* < 0.05). The expression of MMP-3 in the synovium and cartilage tissues of CIA rats was significantly higher than that in control rats, but was significantly lower in the groups treated with PFD and MTX than in CIA rats (Figures 2A–E).

### Bioinformatic Analysis of the Anti-RA Effects of PFD

**FIGURE 3:**
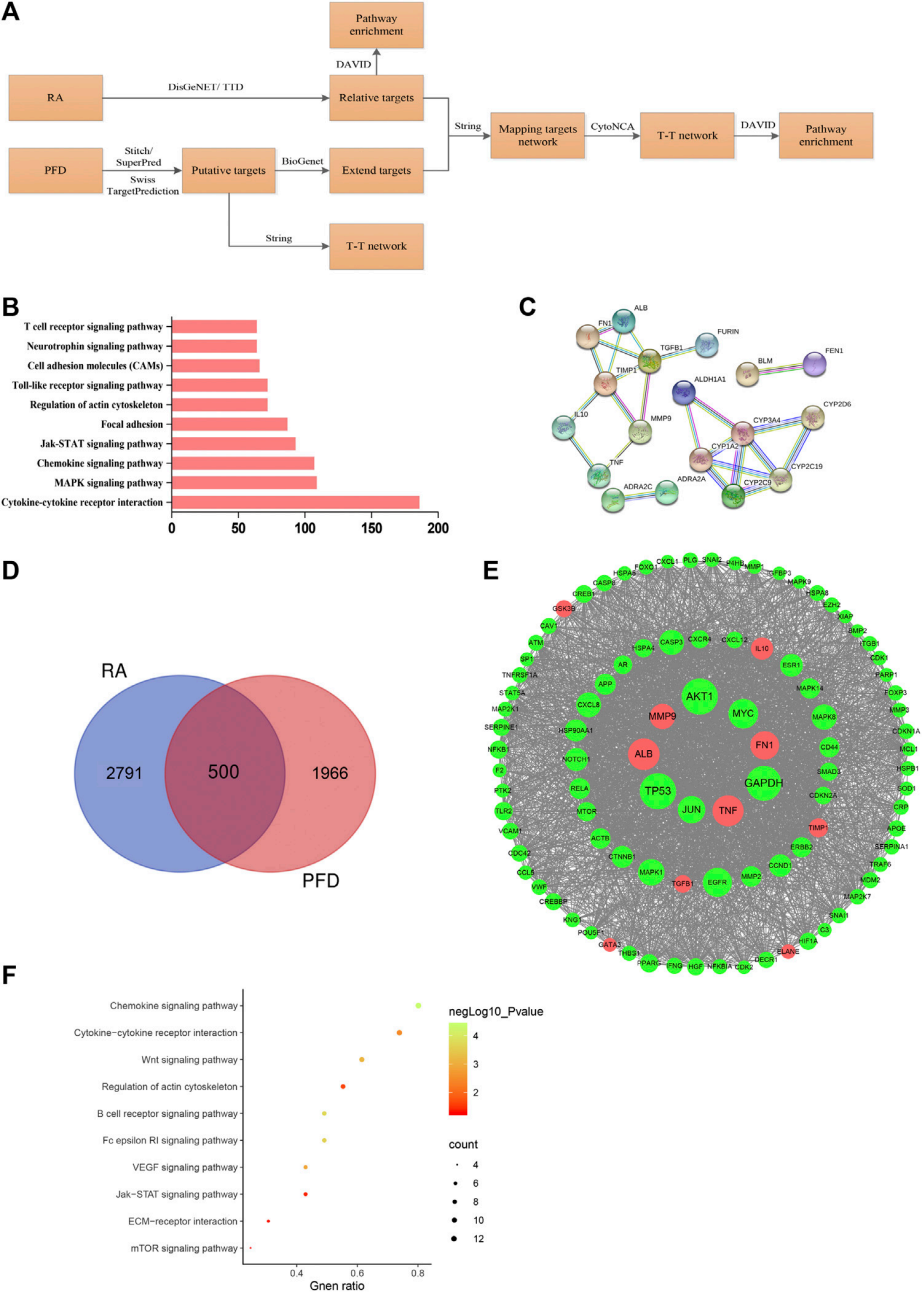
Bioinformatics analysis of the anti-RA effects of PFD **(A)** Summary of the bioinformatics approach for deciphering key pharmacological pathways of the anti-RA effects of PFD. **(B)** Bar chart of the top 10 pathways from a KEGG enrichment analysis of RA-related targets **(C)** PPI network graph of putative PFD targets based on the highest reliability of direct interactions. **(D)** Venn diagram of RA-related targets and putative PFD targets **(E)** PPI network graph of 97 hub nodes based on direct interactions; nodes represents proteins, red nodes represent directs target of PFD, and the node size is proportional to the node degree. **(F)** Scatter plot of the top 10 KEGG pathways based on hub nodes.

Upon searching the Therapeutic Target Database (TTD) and DisGeNET databases, 128 and 2,723 targets related to RA were obtained, respectively, and 2,791 targets were obtained after integration and deduplication. In a KEGG enrichment analysis, the top ten most highly enriched pathways were immune-inflammatory signaling pathways (Figure 3B), consistent with previous findings, indicating that the approach was reliable.

PFD targets were predicted using the STITCH, Swiss TargetPrediction, and SuperPred databases, yielding 10, 9, and 15 targets and a total of 33 targets after integration and deduplication. The predicted targets were imported into the String database to construct a PPI network with adjustments for high reliability. Two important PPI networks were detected (Figure 3C): one including Furin, TGF-β, TIMP1, FN1, MMP-9, IL-10, TNF, and other subnetwork proteins related to inflammation and extracellular matrix function; and another including proteins related to oxidative stress and drug metabolism.

A total of 1966 targets were obtained by extending putative PFD targets, and 500 common targets were shared between PFD and RA (Figure 3D). After using CytoNCA for a topological analysis of targets according to the degree centrality, 97 hub nodes were obtained. Hub nodes were imported into the String database for connection and then into Cytoscape to construct the interaction network (Figure 3E). The AKT node had the highest degree value, indicating that it is an important node. Moreover, previous studies by our group have shown that TNF-α could activate the AKT signaling pathway in MH7A cells (Jia et al., 2015); therefore, follow-up detection of AKT was performed. Overlap between the hub nodes and predicted targets of PFD included 10 loci (marked red), most of which are related to inflammation and extracellular matrix function.

A KEGG enrichment analysis of hub nodes showed that the top ten signaling pathways with statistical significance, which overlapped with enriched signaling pathways related to RA targets, included the JAK/STAT signaling pathway, regulation of actin cytoskeleton, cytokine-cytokine receptor interaction, and the chemokine signaling pathways (Figure 3F). Therefore, we focused on the JAK/STAT pathway in subsequent analyses.

### PFD Suppresses the mRNA and Protein Expression of MMPs and Pro-inflammatory and Angiogenic Cytokines in TNF-α-Stimulated MH7A Cells

**FIGURE 4:**
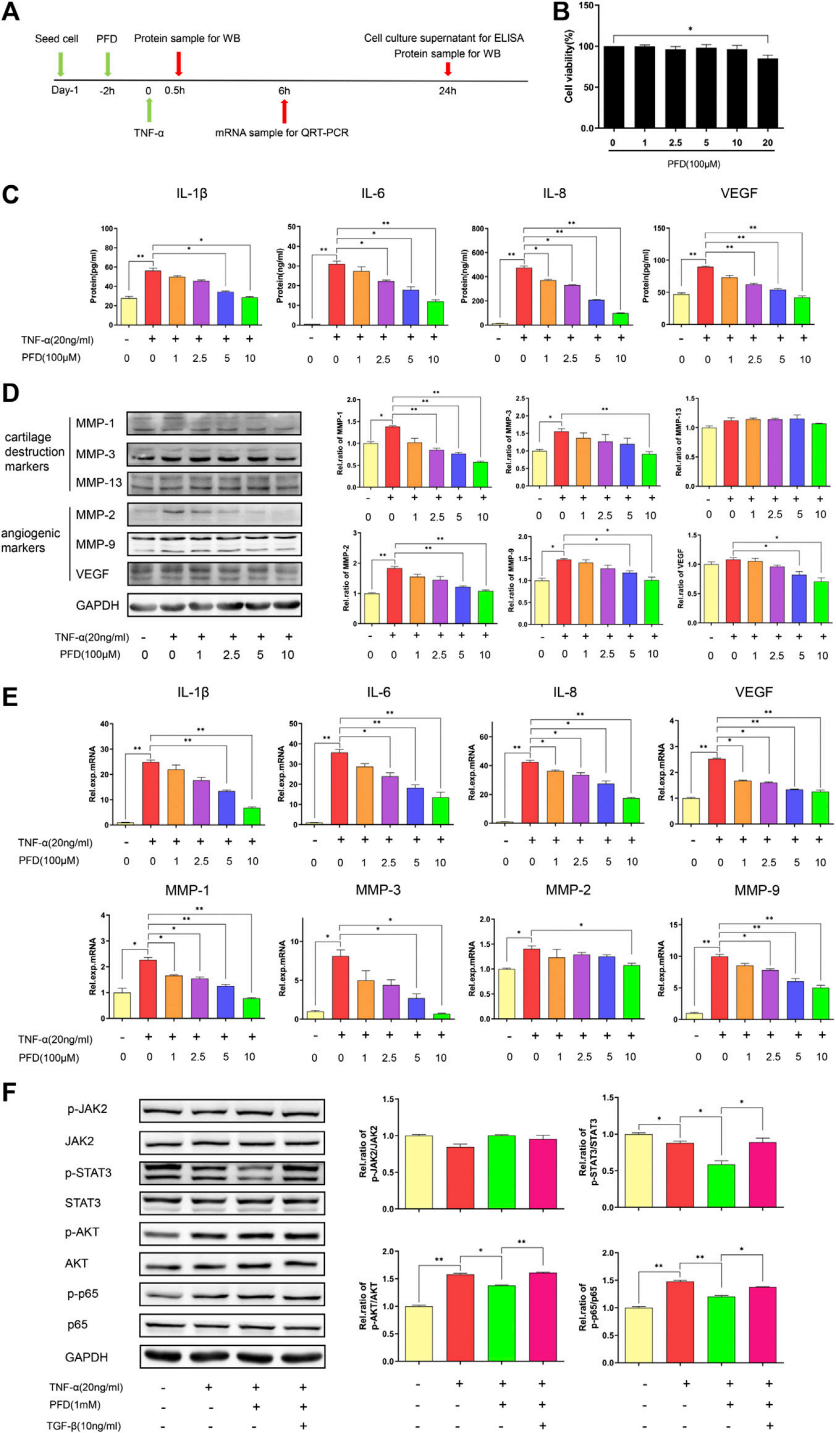
PFD inhibits TNF-α-induced cytokine expression in MH7A cells via multiple signaling pathways **(A)** Flow chart of the experimental design. **(B)** Assay of MH7A cell viability after treatment with various concentrations of PFD for 72 h **(C)** Concentrations of IL-1β, IL-6, IL-8, and VEGF in the culture supernatant. **(D)** Western blotting (MMP-1, MMP-3, MMP-13, MMP-2, MMP-9, and VEGF) **(E)** Relative mRNA expression levels of *IL-1β, IL-6, IL-8, MMP-1, MMP-3, MMP-2, MMP-9,* and *VEGF*. **(F)** MH7A cells were pre-treated with PFD or TGF-β for 2 h prior to TNF-*α* (0.5 h) stimulation, followed by assessments of total and phosphorylated levels of JAK2, STAT3, AKT, and p65 in cell lysates by western blotting. *p < 0.05; **p < 0.01. For the *in vitro* experiments, different colors for the statistical models were uniformly set according to PFD concentration.

The cytotoxicity of PFD on MH7A cells was evaluated using the CCK-8 assay. After 72 h of intervention, PFD at a concentration of <1 mM did not affect the viability of MH7A cells (Figure 4B). Therefore, the four highest non-cytotoxic concentrations of PFD were selected for subsequent experiments. As shown in Figures 4C–E, stimulation of MH7A with TNF-α increased IL1β/6/8, VEGF, and MMP-1/2/3/9 production, while PFD significantly and dose-dependently decreased the expression levels of the above-mentioned cytokines. Trends in mRNA and protein expression levels were consistent, indicating that PFD regulates cytokine expression at the transcriptional level.

### PFD Inhibits TNFα-Induced Pathway Activation via Multiple Pathways

After TNF-α stimulation, the phosphorylation levels of AKT and p65 increased markedly. PFD treatment downregulated the expression of phosphorylated STAT3, AKT, and p65. After TGF-β intervention, the expression levels of phosphorylated STAT3, AKT, and p65 were effectively increased (Figure 4F). Combined with our previous studies, these results indicated that PFD may inhibit the downstream STAT3, Akt-NF-κB-mediated inflammatory signaling pathway by inhibiting the TGF-β target.

### PFD Inhibited the Migration and Tube Formation of EA.hy926 Cells

**FIGURE 5:**
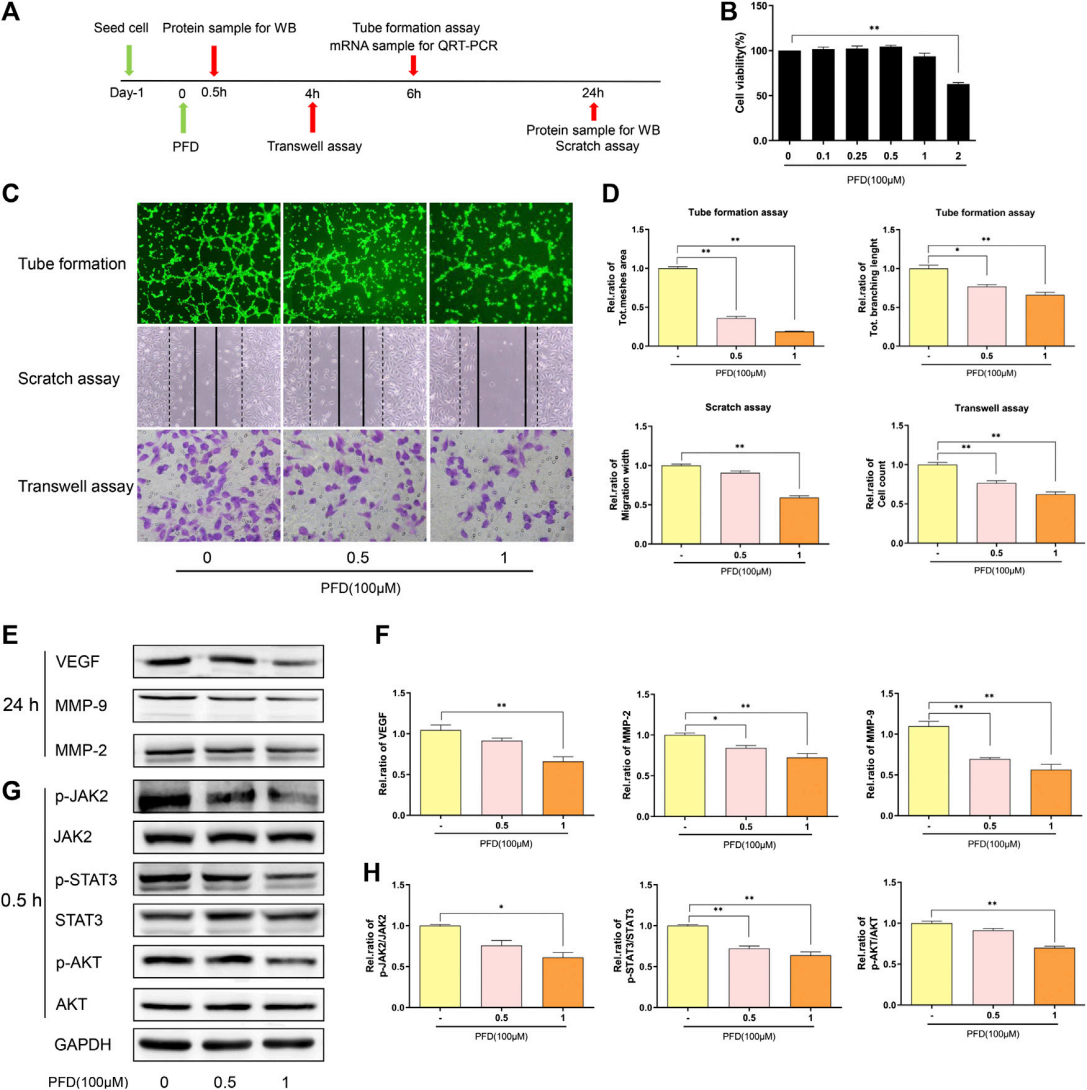
PFD inhibits angiogenesis in EA. hy926 cells **(A)** Flow chart of the experimental design. **(B)** Assay of EA. hy926 cell viability after treatment with various concentrations of PFD for 72 h, **p < 0.01 **(C,D)** EA. hy926 cell tube formation and migration, and results of a semi quantitative analysis, *p < 0.05; **p < 0.01. **(E**,**F)** Western blot results (VEGF, MMP-2, and MMP-9) and summary of a statistical analysis, *p < 0.05; **p < 0.01. **(G,H)** The total and phosphorylated levels of JAK2, STAT3, and AKT were determined by western blotting. *p < 0.05; **p < 0.01. For the *in vitro* experiments, different colors for the statistical models were uniformly set according to PFD concentration.

First, using the CCK-8 assay to detect the cytotoxicity of PFD against EA. hy926 cells (Figure 5B), 100 μm and 50 µm concentrations of PFD were selected for subsequent experiments. PFD effectively inhibited tube formation and significantly inhibited the recovery of the scratch width and cell migration in the Transwell (Figures 5C,D) in a dose-dependent manner. As shown in Figure 5E, PFD significantly decreased the expression of VEGF and MMP-2/9, at the protein level, consistent with the observed inhibition of tube formation and migration. These results indicate that PFD may inhibit the tube formation and migration of EA. hy926 cells by downregulating the expression of angiogenic factors. To further investigate the mechanisms by which PFD inhibits the production of VEGF and MMP-2/9, based on bioinformatics predictions, we examined the JAK2/STAT3 and AKT-mediated signaling pathways. PFD significantly inhibited the expression of phosphorylated JAK2/STAT3 and AKT in a dose-dependent manner (Figure 5E).

## Discussion

The synthetic small molecule inhibitor PFD has inhibitory effects on pro-inflammatory cytokines and matrix metalloproteinases, which may be beneficial for RA treatment. Clinical studies have shown that PFD alleviates pain in patients with RA (Pesce et al., 2004). However, pathological changes in response to PFD have not been evaluated in animal models of RA. In this study, we provide the first evidence that PFD effectively inhibits synovial inflammation and joint destruction in CIA rats, strongly supporting the clinical research of Pesce et al. (2004).

Using the CIA model, we found that PFD effectively inhibited joint synovial inflammation and relieved joint swelling in CIA rats, which may explain its beneficial effects on clinical pain in RA. Compared to MTX, PFD has fewer side effects, and its clinical application is safe and reliable. RA is often accompanied by pulmonary fibrosis in patients, and PFD could effectively alleviate both these pathologies in its treatment of RA-associated pulmonary fibrosis. Thus, PFD appears to be a potential drug for the treatment of RA, with broad prospects for clinical application. Studies have shown that FlSs can produce MMPs and inflammatory cytokines, which play a critical role in cartilage destruction and inflammatory infiltration in RA. Therefore, the inhibition of FlS activity may alleviate RA symptoms. MMPs can cause matrix degradation and cartilage destruction and accelerate the pathological progression of RA joints, and inhibition of MMPs can effectively reduce cartilage erosion (Korb-Pap et al., 2016). In this study, immunohistochemistry confirmed that PFD reduces MMP-3 in the joints of CIA rats, consistent with the trend in bone destruction observed by histology, indicating that PFD protects articular cartilage by decreasing MMP-3 expression. Local chronic hypoxia and inflammation may lead to pannus formation, which can promote inflammatory infiltration and structural damage (Jakobiec et al., 2014; MacDonald et al., 2018). VEGF is an important regulator of angiogenesis (Sene et al., 2015; Melincovici et al., 2018), which could promote endothelial cell migration and vascular development. In our study, immunohistochemical staining showed that PFD effectively reduced VEGF expression in the joint, indicating that it may reduce synovial pannus hyperplasia.

A bioinformatic analysis further suggested that PFD exerts anti-RA effects by inhibiting the expression of inflammatory cytokines and cell migration. A KEGG enrichment analysis indicated that PFD may protect against RA via the JAK2/STAT3, PI3K/AKT, and NF-κB pathways. Therefore, we detected inflammatory cytokines and MMP proteins and evaluated the effects of PFD on these pathways *in vitro*. MH7A cells were stimulated with TNF-α to simulate synovial inflammation and the local microenvironment, and PFD effectively reduced the expression of pro-inflammatory, angiogenic, and chondrogenic destruction-related cytokines, such as IL1β/6/8, VEGF, and MMP-1/2/3/9 (McInnes et al., 2016; Liu et al., 2018; Malemud, 2019). Downregulation of the AKT pathway can effectively reduce proliferation, migration, and inflammatory cytokine expression in synovial fibroblasts (Mainz et al., 2016; Song et al., 2019). Blocking the STAT3 signaling pathway can effectively inhibit synovial inflammation in RA (Gao et al., 2015). Our results indicate that PFD may inhibit inflammatory activity in MH7A cells and alleviate the local inflammatory microenvironment by inhibiting the STAT3 and AKT/NF-κB signaling pathways. The proliferation, migration, and tube formation of vascular endothelial cells are the key steps in the early stage of angiogenesis (Pardali et al., 2010). Migration and tube formation of endothelial cells are regulated by VEGF and MMPs, among which MMP-2 and MMP-9 are type IV collagenases, which can decompose the ECM and promote cell migration (Liu et al., 2018). Targeting VEGF and MMP2/9 could effectively alleviate RA symptoms (Li et al., 2018; Li et al., 2019). We found that PFD effectively reduced the expression of VEGF and MMP-2/9, and weakened the migration of EA. hy926 cells, resulting in the inhibition of angiogenesis. The activation of STAT3 pathway could regulate the expression of VEGF and MMPs and promote blood vessel formation (Banerjee and Resat, 2016). The activation of phosphatidylinositol 3-kinase (PI3K)/Akt is essential for cell proliferation and migration during angiogenesis (Ruan and Kazlauskas, 2012). Our results showed that PFD may inhibit angiogenesis in EA. hy926 cells by inhibiting the activity of the JAK2/STAT3 and PI3K/AKT pathways.

## Conclusion

**FIGURE 6:**
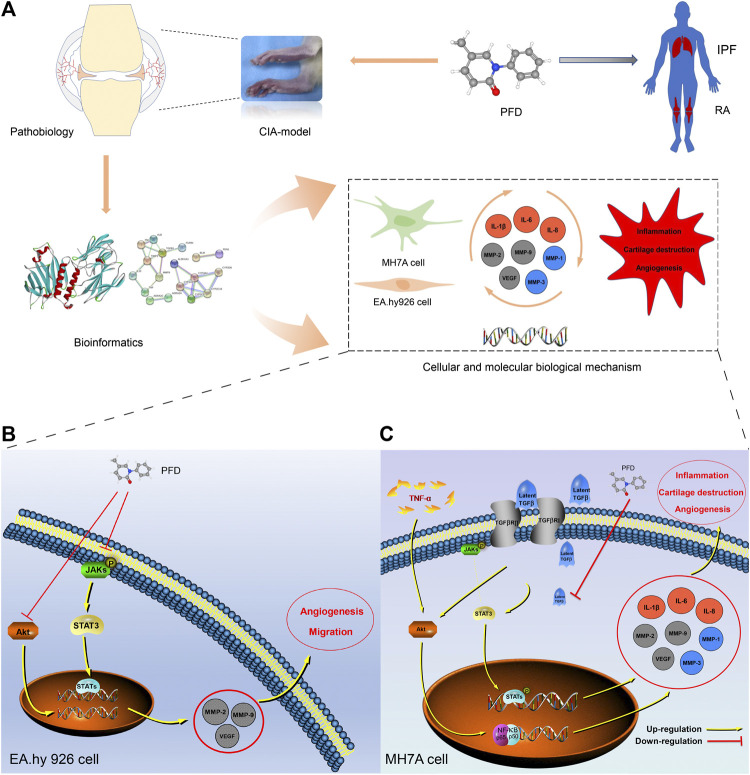
Mechanism underlying the effects of PFD against RA **(A)** Technical strategy for deciphering the key determinants of the anti-RA effects of PFD, IPF: idiopathic pulmonary fibrosis. **(B)** PFD alleviated angiogenesis of EA. hy926 cells by inhibiting multiple pathways. **(C)** PFD alleviated TNF-*α*-induced inflammation, cartilage degradation, and angiogenesis by inhibiting multiple pathways in MH7A cells. Orange circles: inflammatory markers; Gray circles: angiogenic markers; Blue circles: cartilage destruction markers.

In summary, our study demonstrated, for the first time, that PFD may effectively alleviate symptoms and delay pathological processes in CIA rats by inhibiting inflammation and angiogenesis through multiple pathways (Figure 6). These findings clearly support the clinical application of PFD for the treatment of RA. More broadly, our findings, based on comprehensive *in vitro*, *in vivo*, and bioinformatics analyses, contribute to the recent literature supporting the use of therapeutic strategies targeting synovial inflammation and angiogenesis in RA.

## Data Availability

The raw data supporting the conclusions of this article will be made available by the authors, without undue reservation, to any qualified researcher.
